# Message Framing Analysis: Recommendations for Dental Health PSAs

**DOI:** 10.3290/j.ohpd.c_2076

**Published:** 2025-07-04

**Authors:** Meyassara Samman

**Affiliations:** a Meyassara Samman Assistant Professor, Department of Dental Public Health, Faculty of Dentistry, King Abdulaziz University, Jeddah, Saudi Arabia. Conceptualisation, data extraction, analysis, validation, visualisation, writing original draft, reviewing and editing.; b

**Keywords:** advertising, dental public health, message framing, public service announcements, YouTube

## Abstract

**Purpose:**

Dental caries is a prevalent global public health issue, influenced by poor oral hygiene and high sugar consumption. Media, particularly advertising, plays a crucial role in shaping these behaviours. While message framing has been widely studied in commercial advertising, little is known about its application in dental health public service announcements (PSAs). This study aims to analyse dental health PSAs and commercial advertisements (ads) through the lens of message framing theory to identify how different framing strategies can enhance audience engagement.

**Methods:**

A systematic YouTube search was conducted to identify advertisements in four categories: dental products, chocolates, sodas, and dental health PSAs. The first three advertisements from each category were analysed. Each ad was evaluated based on its promise, support elements, and core values. The framing strategies used in commercial advertisements were compared with those used in dental health PSAs.

**Results:**

The analysis revealed that commercial advertisements for dental products, chocolates, and sodas employed diverse framing strategies focused on emotional appeals, aligning their messages with values such as success, freedom, and social acceptance. In contrast, dental health PSAs consistently emphasised health related messages supported by educational content.

**Conclusions:**

Dental health PSAs often fail to connect with the public due to their focus on rational, health based appeals, whereas commercial advertisements more effectively engage audiences by framing messages around personal or social values. Reframing dental health PSAs to incorporate emotional drivers and align with audience values, such as freedom, social connection, and success, could improve their effectiveness in promoting oral health behaviours.

Dental caries is a prevalent and multifactorial chronic disease that affects a significant portion of the global population. It is one of the most common noncommunicable diseases worldwide, affecting people of all ages and socioeconomic backgrounds.^1,12^ Approximately 3.5 billion people globally experience one or more untreated carious lesions. Caries in primary teeth affects about 43% of the population, while caries in permanent teeth is estimated at 29%.^4^ In the United States, 35% of children have dental caries, with 18% having untreated decay.^8^ Additionally, 91% of adults have experienced dental caries, and 27% have untreated carious lesions.^11^ While advancements in dental technologies and preventive care have been made, dental caries remain a major public health issue, exacerbated by individual behaviours and lifestyle choices, including poor oral hygiene, high sugar consumption, and a lack of preventive measures.^12,13^

Media, particularly television and online platforms, play a crucial role in shaping health related behaviours.^24^ Advertisements (ads) promoting sugary foods and beverages are widespread and often encourage unhealthy dietary choices that may negatively impact oral health.^9^ Platforms like YouTube, X (formerly Twitter), Facebook, and Instagram have become major sources of influence, especially among younger audiences. These platforms not only host advertisements but also enable user generated content and influencer campaigns, which further impact public perceptions and behaviours related to oral health. However, dental health public service announcements (PSAs) remain scarce across these media channels, and when aired, they often focus solely on health related information, failing to resonate with broader public values such as freedom, success, or social connection.

Message framing is a communication method that emphasises specific aspects of a message to influence an audience’s perceptions and actions. It is based on the idea that the way information is presented has a significant effect on decision making and behavioural outcomes.^20^ Positive frames highlight the benefits of a certain behaviour, whereas negative frames highlight the consequences of inaction. Effective framing considers the target audience’s values, beliefs, and emotional drivers to craft messages that resonate on a deeper, more personal level.^3,23^

Message framing in dentistry has been studied through various communication channels, including text and web-based methods. Research has demonstrated its effectiveness in improving oral health knowledge, attitudes, intentions, self efficacy, and practices across different populations. Studies have explored both gain and lossframed messages, with some indicating equal effectiveness and others suggesting that loss framed messages may be more impactful in certain contexts.^5,7,18,19^ Although message framing has demonstrated potential in enhancing oral health promotion through various mediums, its application in dental health PSAs remains an area for further exploration. The optimal framing strategy may vary depending on the target population and specific oral health behaviour being promoted, highlighting the need for tailored approaches in dental health communication.

This study analyzes and compares advertisements for dental products, soda, chocolate, and dental health PSAs using message framing as a framework. By evaluating how promise, support, and core values are framed in these ads, we aim to provide recommendations for enhancing the effectiveness of dental health PSAs.

## METHODS

This study conducted a qualitative content analysis of advertisements, and employed a systematic approach to identify and analyze advertisements in four categories: dental products, soda, chocolates, and dental health PSAs. These categories were chosen to provide a diverse range of advertisement types for analysis, allowing for a comparison of framing strategies across products associated with oral health (dental products) and behaviours linked to dental caries (chocolates and sodas), as well as public health campaigns (dental health PSAs).

YouTube was chosen as the primary platform for advertisement retrieval due to its accessibility and comprehensive repository of both commercial and public health advertisements. A systematic search was conducted, using specific keywords adapted to each category: ‘Toothpaste advertisements USA’ for dental products, ‘Chocolates advertisements USA’ for chocolates, ‘Soda advertisements USA’ for sodas, and ‘Dental public health PSA USA’ for dental health PSAs.

The first three advertisements from each search were selected for analysis. Advertisements were included if they were in English and excluded if they were part of a movie trailer or not productspecific. The selection of three advertisements per category was based on the exploratory nature of the study, prioritising feasibility and the opportunity for an in depth qualitative analysis. This sample size was not intended to generalise the findings, but rather to provide initial insights into message framing strategies.

The advertisements analysis utilised a deductive coding approach, guided by predefined categories based on message framing theory, with a focus on three main components:

**Promise:** The key message or benefit the advertisement promises to deliver.
**Support:** The elements (eg, visuals, music, celebrities) that reinforce the promise.
**Core values:** The fundamental values or ideals that the advertisement seeks to evoke in its audience, and the message aligns with.

These framing strategies were selected because they comprehensively capture the elements of effective advertising and enable meaningful comparisons between commercial advertisements and dental health PSAs.

## RESULTS

### Dental Products

The dental product advertisements demonstrate diverse message framing strategies, each focusing on unique core values. The Oral-B ad highlights personal success, utilising a celebrity endorsement to associate oral care with achievement. Colgate, on the other hand, frames its message around social responsibility by promoting water conservation as a part of maintaining oral hygiene. Sensodyne takes a different approach by emphasising comfort, offering scientific support to address tooth sensitivity. These advertisements are designed to resonate with distinct audience motivations, such as success, altruism, and comfort (Table 1).

**Table 1:** Message framing and core values in dental products ads

	Ad 1 (Oral-B®)	Ad 2 (Colgate®)	Ad 3 (Sensodyne®)
**Promise**	Engaging in the activities you love	Saving water will positively impact others	Less tooth sensitivity, enabling enjoyment of favorite foods
**Support**	Featuring global pop star Shakira in a recording studio	Describes water conservation in daily activities (washing fruits, drinking, etc.)	Detailed scientific explanation of how Sensodyne reduces sensitivity
**Core value**	Success	Altruism	Comfort


### Chocolates

Chocolate advertisements often leverage emotional appeals to connect with their audience’s values. Dove promotes the idea of personal freedom, encouraging viewers to fully embrace life, whereas Magnum focuses on empowerment, using bold imagery to convey control and strength. In contrast, Snickers targets social acceptance, portraying its product as a means of enhancing group experiences and promoting a sense of belonging. Each ad taps into emotional drivers – freedom, empowerment, and social connection – to engage with its audience effectively (Table 2).

**Table 2:** Message framing and core values in chocolate ads

	Ad 1 (Dove®)	Ad 2 (Magnum®)	Ad 3 (Snickers®)
**Promise**	Living a life without regrets	Take control of life with bold indulgence	Enhancing social interactions through shared enjoyment
**Support**	Features an average woman enhancing her routine with Dove chocolate to the tune of song ‘No regrets’ Ad quote: live each day as if it’s the only one Storytelling	Fierce animals and vibrant visuals Ad quote: ‘Release the beast’	A group of friends engaging in fun activities Storytelling
**Core value**	Freedom	Personal control	Social acceptance


### Sodas

Coca Cola advertisements employ a variety of message framing techniques to evoke diverse emotional responses. One ad emphasises fun and togetherness through the theme of sharing, another showcases technological innovation to create an engaging experience, and a third focuses on strengthening family bonds, particularly between siblings. These advertisements use specific creative elements to align the brand with social interactions, innovation, and familial relationships (Table 3).

**Table 3:** Message framing and core values in soda ads

	Ad 1 (Coca Cola®)	Ad 2 (Coca Cola®)	Ad 3 (Coca Cola®)
**Promise**	Enhancing fun and good times with friends	A refreshing, innovative experience	Strengthening family relationships, especially among siblings
**Support**	Music Friends enjoying activities together	An extensive campaign featuring interactive apps, posters, and media that encourage engagement with the product	Music Depicts typical sibling dynamics and moments of bonding
**Core value**	Sharing and connection	Innovation	Family connection


### Dental Health PSAs

The dental health PSAs all focus on promoting health, with each ad framing its message through different forms of support. The Florida ad uses cartoons and education to engage children, the New Jersey ad targets caregivers by offering guidance from an actress, and the 2 Minutes Campaign uses a relatable family scenario to encourage preventive action. Despite these differences, the core value of health is consistently emphasised across all the advertisements (Table 4).

**Table 4:** Message framing and core values in dental health PSAs

	Ad 1 (Florida department of health)	Ad 2 (New Jersey dental association)	Ad 3 (2 minutes campaign)
**Promise**	Promoting healthy teeth and reducing caries	Reducing caries through proper dental care	Brushing for two minutes will prevent tooth pain in children
**Support**	Cartoon characters explaining caries prevention	An actress explaining how parents can prevent caries in children	Shows a father and son brushing together
**Core value**	Health	Health	Health


## DISCUSSION

The analysis highlights the contrast between commercial advertisements and dental health PSAs. Commercial advertisements typically frame their messages around broader emotional appeals and values such as freedom, success, and social connection, whereas dental health PSAs tend to focus solely on health outcomes and support them with information, knowledge, and statistics.

Health is essential to public health practitioners, dentists, and physicians. In contrast, most of the general population considers health a secondary outcome compared to personal freedom and individual rights.^18^ Although health is an important outcome, public health messages must be reframed to appeal to the general population.

Message framing emphasises the importance of presenting information in ways that resonate with the target audience’s values and emotional drivers. The commercial advertisements reviewed in this study demonstrate how effectively framing a product as a means to achieve personal or social goals can engage audiences at a deeper level. In contrast, dental health PSAs often assume rational behaviour, focusing on health information rather than emotional engagement. These PSAs typically use the health belief model (HBM), expecting that individuals will logically weigh the costs and benefits of dental care and act in their best interest. However, the limitation of the HBM is that it is an individual focused model, which, even if successful in changing personal behaviours, may not lead to broader population level impacts; a key objective of PSAs.^17^

The HBM does not account for emotional or social influences, which are often critical in shaping health behaviours. For example, commercial advertisements leverage emotional appeal and societal values such as freedom, success, or connection to drive behaviour change. These elements are largely absent in HBM focused PSAs, potentially limiting their ability to effectively engage audiences. By adopting alternative frameworks, such as Social Cognitive Theory or the Theory of Planned Behaviour, future PSAs could integrate emotional and social dimensions, fostering stronger connections with the target audience and enhancing public health outcomes.

Research has shown that decision making is rarely purely rational; emotions, biases, and framing play significant roles in influencing behaviours.^19^ While studying youth to create anti-tobacco (Truth) campaign, Florida’s health department found that what drives youth to smoke tobacco has nothing to do with rational decision making. Rather, their behaviour is driven by emotions.^14^

While emotional appeals are effective in engaging the general public, rational, healthbased messages may be more suitable for individuals who are already motivated by health concerns. For example, dental professionals, caregivers, and high risk populations (eg, individuals with diabetes or elderly adults) may respond better to evidence based messages that provide clear, actionable health information.

This study did not incorporate quantitative data or numbers to assess the effectiveness of the advertisements and PSAs analysed, limiting our ability to draw conclusions about their impact on behavioural change. Furthermore, the number of available dental health PSAs on YouTube was limited, possibly underrepresenting broader efforts in dental public health media. The analysis focused primarily on the elements of message framing, such as promise, support, and core values, without considering other critical aspects of advertisement analysis, such as production quality, viewer engagement metrics, or the frequency of ad exposure. Additionally, the study did not explore the demographic characteristics of the audience, which could influence how messages are received and processed.

The study’s exploratory nature and the limited sample size of three advertisements per category pose limitations to generalisability. We acknowledge that the chosen keywords, such as ‘toothpaste advertisements USA’, may have limited the scope of the analysis by excluding other dental products like toothbrushes or dental floss. While this ensured feasibility and consistency, it also reduced the comprehensiveness of the findings. Future research with larger sample sizes and quantitative methods is needed to validate and expand upon these findings. In addition, researchers should expand the range of keywords to capture a broader spectrum of dental advertisements and provide more generalisable insights. Experimental studies are also needed to test the effectiveness of different framing strategies in dental health PSAs. Controlled experiments could compare audience responses to emotional versus rational appeals, providing empirical evidence to inform and optimise public health messaging strategies.

Despite its limitations, this study has several key strengths. First, this study is one of the few to examine dental health PSAs compared with commercial advertisements, offering valuable insights into the differences in message framing strategies across these domains. By using a structured framework to analyze core values and emotional appeals, this study highlights the potential for improving the effectiveness of dental health PSAs through better alignment with audience motivations.

### Recommendations

Public health campaigns aiming to enhance the effectiveness of dental health PSAs should consider reframing their messages to incorporate emotional and value driven appeals, similar to the strategies observed in commercial advertisements. Aligning health messages with audience values; such as personal freedom, social connection, or success, can significantly enhance engagement and behavioural impact. To be more effective, future PSAs should move beyond traditional health focused narratives and adopt emotionally resonant techniques that align with the core motivations of the target audience. Incorporating emotional storytelling, relatable narratives, and aspirational themes can make PSAs more compelling. Additionally, leveraging influencers or testimonials, similar to commercial marketing strategies, can further strengthen emotional engagement and audience connection.

To improve dental health PSAs, adopting marketing principles can be highly beneficial. The key steps are as follows:

Understanding the audience: Understanding what the audience wants, needs, and expects is crucial, along with identifying potential barriers to behavioural change.
Shift the approach: Instead of assuming that knowledge will drive change (knowledge → attitude → behaviour), focus on motivating behaviour first (behaviour → attitude → knowledge).
Frame the behaviour: Package the message in a way that resonates with the audience.^21^

Lyle Sussman defined a frame as a tool that directs a reader’s or listener’s attention toward specific aspects of a message, just as a picture frame draws focus to what is within its borders. Framing is particularly effective in addressing irrational decision making,^6^ and significantly influences behavioural change.^10^ Sussman proposed a four step model for building an effective frame:

Define your objective.
Understanding the strengths, weaknesses, opportunities, and threats (SWOT analysis) of the population.
The core values, beliefs, and principles driving the target audience’s behaviours are identified.
Craft a message that integrates these elements.^22^

Public service announcements (PSAs) can use storytelling techniques that depict relatable scenarios, such as a parent child interaction emphasising the joy of healthy smiles, or feature aspirational themes of success linked to oral health.

## CONCLUSION

The use of message framing reveals that dental health PSAs can be more effective by framing messages around the emotional and personal values of their target audience, which is similar to the strategies employed in commercial advertisements. By aligning PSAs with values such as freedom, success, and social connection, public health campaigns can improve engagement and impact, leading to better oral health outcomes.

### Declarations

**Ethics approval:** This research did not require IRB approval because the data analysed in this study consisted of publicly available YouTube videos.
